# Supplementary material for: An automated, high-resolution phenotypic assay for adult Brugia malayi and microfilaria
Source: Sci Rep. 2024 Jun 7;14:13176. doi: 10.1038/s41598-024-62692-x (PMC11161659; doi:10.1038/s41598-024-62692-x)
Supplement: Supplementary file 1 — Supplementary Information 1. [file 41598_2024_62692_MOESM1_ESM.docx]

Supplementary Materials for

**Title: An automated, high-resolution phenotypic assay for adult *Brugia malayi* and microfilaria**

Authors: Upender Kalwa, Yunsoo Park, Michael J. Kimber, Santosh Pandey

Corresponding author: [pandey@iastate.edu](mailto:pandey@iastate.edu)

Movies of tracked body shapes of adult *B. malayi* and microfilaria (mf)

The software program tracks the body shape of the *B. malayi* through each image frame of recorded videos. The five movies (Movies S1-S5) are the outputs of the software program for different *B. malayi* mf worms. The next movie (Movies S6) is the software output for adult *B. malayi*.

Movie S1

Tracked worm boundary and midline skeleton in movie 1 for *B. malayi* mf worms.

**Movie S2**

Tracked worm boundary and midline skeleton in movie 2 for *B. malayi* mf worms.

**Movie S3**

Tracked worm boundary and midline skeleton in movie 3 for *B. malayi* mf worms.

**Movie S4**

Tracked worm boundary and midline skeleton in movie 4 for *B. malayi* mf worms.

**Movie S5**

Tracked worm boundary and midline skeleton in movie 5 for *B. malayi* mf worms.

Movie S6

Tracked worm body parameters in movie 6 for adult *B. malayi* in control.

Movie S7

Tracked worm body parameters in movie 7 for adult *B. malayi* in 100 µM albendazole.

Movie S8

Tracked worm body parameters in movie 8 for adult *B. malayi* in 10 µM ivermectin.
